# Community Knowledge, Perceptions, and Practices Associated with Urogenital Schistosomiasis among School-Aged Children in Zanzibar, United Republic of Tanzania

**DOI:** 10.1371/journal.pntd.0004814

**Published:** 2016-07-11

**Authors:** Bobbie Person, Said M. Ali, Faiza M. A’Kadir, Jamal N. Ali, Ulfat A. Mohammed, Khalfan A. Mohammed, David Rollinson, Stefanie Knopp

**Affiliations:** 1 The Schistosomiasis Consortium for Operational Research and Evaluation, University of Georgia, Atlanta, Georgia, United States of America; 2 Public Health Laboratory—Ivo de Carneri, Chake Chake, Pemba, United Republic of Tanzania; 3 Zanzibar Neglected Tropical Diseases Control Program, Ministry of Health, Zanzibar Town, Unguja, United Republic of Tanzania; 4 Wolfson Wellcome Biomedical Laboratories, Department of Life Sciences, Natural History Museum, London, United Kingdom; 5 Swiss Tropical and Public Health Institute, Basel, Switzerland; 6 University of Basel, Basel, Switzerland; London School of Hygiene and Tropical Medicine, UNITED KINGDOM

## Abstract

**Background:**

On the Zanzibar islands, United Republic of Tanzania, elimination of urogenital schistosomiasis is strived for in the coming years. This qualitative study aimed to better understand community knowledge, perceptions, and practices associated with schistosomiasis among school-aged children on Unguja and Pemba islands, in order to inform the development of behavior change interventions contributing to eliminate urogenital schistosomiasis.

**Methodology:**

In 2011, we conducted 35 children’s discussion groups, 41 in-depth interviews with parents and teachers, and 5 focus group discussions with community members in Zanzibar. Using a modified-grounded theory approach, we transcribed and coded the narrative data followed by thematic analysis of the emergent themes.

**Principal Findings:**

Urogenital schistosomiasis is a common experience among children in Zanzibar and typically considered a boys’ disease. Children engage in multiple high-risk behaviors for acquiring schistosomiasis because of poor knowledge on disease transmission, lack of understanding on severity of disease-associated consequences, and lack of alternative options for water related activities of daily living and recreational play. Local primary school teachers had little to no training about the disease and no teaching tools or materials for students.

**Conclusions/Significance:**

Conducting activities in open natural freshwater contaminated by *S*. *haematobium* larvae compromises the health of school-aged children in Zanzibar. The perception of urogenital schistosomiasis as a minor illness rather than a serious threat to a child’s well-being contributes to the spread of disease. Understanding community perceptions of disease along with the barriers and facilitators to risk reduction behaviors among children can inform health promotion activities, campaigns, and programs for the prevention, control, and elimination of urogenital schistosomiasis in Zanzibar.

## Author Summary

On the Zanzibar islands, United Republic of Tanzania, elimination of urogenital schistosomiasis, a disease caused by infection with a blood fluke (*Schistosoma haematobium*), locally known as kichocho, is strived for in the coming years. This study used qualitative research methods of focus groups and in-depth interviews with adults, and group discussions with school-aged children to explore (i) knowledge and perceptions of kichocho transmission, (ii) specific behaviors among children in Zanzibar that put them at risk for acquiring infections with the kichocho parasite, (iii) symptoms of kichocho along with personal health-seeking behaviors and treatment strategies, and finally (iv) ideas for preventing kichocho in children. We found that there was little available formal education about disease transmission, which contributed to myths and misperceptions about routes of transmissions, causes, and treatment of kichocho. School-aged children regularly exposed themselves to contaminated natural, open freshwater bodies through recreational and domestic activities of daily life. Kichocho was often wrongly viewed as an infection with an intestinal worm of little significance, rarely associated with severe health consequences, and with little to no disease stigma. Local primary school teachers had little to no training about the disease and no teaching tools or materials for students. The findings add valuable insights into how current knowledge, perceptions, and practices impede optimal disease prevention and control and highlight the necessity for a community tailored behavioral intervention to interrupt transmission of urogenital schistosomiasis.

## Introduction

Schistosomiasis is a debilitating disease that affects poor and deprived population groups, especially in rural Africa [1]. The global impact is enormous with more than 200 million people infected with blood flukes of the genus *Schistosoma* [2]. Urogenital schistosomiasis, caused by *S*. *haematobium*, can include acute illness such as blood in urine (hematuria) and anemia in children, while fibrosis of the bladder and ureter, and kidney damage can occur as infections persist [3,4]. Bladder cancer can be a complication in adults and female genital schistosomiasis may be associated with increased risk of human immunodeficiency virus (HIV) infection [5–9].

Over the past decades, programs to reduce the morbidity caused by schistosomiasis have been implemented in many endemic countries and the number of people who received treatment with praziquantel has increased annually [10]. In order to eliminate schistosomiasis as a public health problem and to interrupt transmission in areas where morbidity control has been achieved, the World Health Organization (WHO) and other institutions and stakeholders are advocating the intensified use of integrated schistosomiasis control approaches [7,11–14]. Health education and interventions based on social and behavioral science triggering behavior change, in addition to regular preventive chemotherapy with praziquantel, are likely to become a key component of future elimination efforts [12,13,15].

Behavior change in humans requires close interaction with the at-risk population [16]. To change risk behaviors, enhance the interruption of disease transmission, and finally eliminate schistosomiasis particularly in children, innovative, community-tailored approaches are needed. Understanding the community is critical to creating effective behavioral interventions promoting the adoption of protective behaviors and reducing risk behaviors [16,17]. Community participatory processes are fundamental to understanding the community’s current knowledge, perceptions, attitudes, and behaviors as well as motivators and barriers to behavior change [18]. Community participation can also create ownership of public health initiatives, which is often viewed as fundamental for the success of population-based health outcomes [16,18]. People are experts about the communities they live in and have many different ways of knowing and gathering information.

On the Zanzibar islands, United Republic of Tanzania, elimination of urogenital schistosomiasis is strived for in the coming years. In the frame of a randomized operational research trial implemented on Unguja and Pemba islands from 2012 till 2017, the impact of behavior change interventions in addition to biannual praziquantel treatment on the prevalence and intensity of *S*. *haematobium* infections is assessed [19–21]. Here we present results of the qualitative formative research that was implemented in 2011 to inform, along with future participatory community co-design workshops, the development of a community-tailored behavioral change intervention that might help to eliminate urogenital schistosomiasis in Zanzibar [19].

## Methods

### Ethics Statement

In 2011, the National Centre for Emerging Zoonotic Diseases (NCEZID) of the Centers for Disease Control and Prevention (CDC) received and approved the qualitative formative research protocol for Zanzibar (NCEZID Tracking Number: 103111BP) to go forward to the CDC Human Research Protection Office (HRPO) and Institutional Review Board (IRB) for review. The HRPO and IRB determined that the project activities were exempt under regulation 45 CFR 46.101(b)(2) and issued a written waiver. The full study protocol of the “Study and implementation of schistosomiasis elimination in Zanzibar (Unguja and Pemba islands) using an integrated approach” received additional ethical approval from the Zanzibar Medical Research Ethics Committee in Zanzibar, United republic of Tanzania (reference no. ZAMREC 0003/Sept/011), the “Ethikkommission beider Basel” (EKBB) in Basel, Switzerland (reference no. 236/11) and the IRB of the University of Georgia, in Athens, Georgia, United States of America (project no. 2012-10138-0). The study is registered at the International Standard Randomized Controlled Trial Number Register (ISRCTN48837681). The data collection was conducted with support from the CDC in Atlanta, Georgia, United States of America. The CDC HRPO and IRB approved the informed consent process conducted with all participants, who took part in student group discussions and interviews. Due to a limited ability of participants to read and write the informed consent was available in both English and Kiswahili, the local language, and read aloud by trained bilingual research staff. Participants provided a verbal consent, with the consent acknowledged with the signature on the informed consent document of a witness present at the time [22,23]. Research staff reviewed the consent procedure and all consent forms to ensure compliance with the process. In case of children below the age of 18 years, their parents or legal guardians provided written informed consent for their participation.

### Study Area and Population

This qualitative inquiry was conducted including school-aged children, parents, teachers, and community leaders from seven small administrative areas, called shehias, on the islands of Unguja and Pemba from July till September 2011. The islands of Unguja and Pemba have an estimated combined population of around 1.3 million people and the main industries are spices, raffia, and tourism [24,25]. More than 99 percent of Zanzibar's population is Muslim. Urogenital schistosomiasis constituted a considerable public health problem on both islands in the past century [26–28], but regular treatment of the at risk population with praziquantel reduced *S*. *haematobium* prevalences and intensities [29–31]. In 2012, the baseline survey of the “Study and implementation of schistosomiasis elimination in Zanzibar (Unguja and Pemba islands) using an integrated approach” revealed an overall prevalence of 4.3% and 8.9% in schoolchildren from Unguja and Pemba, respectively [20].

### Research Team

The field team consisted of a senior social scientist from the CDC and seven Kiswahili and English speaking research assistants from the Ministry of Health, Department of Neglected Tropical Diseases, and the Ministry of Education, Department of Health Education, in Unguja, and three research assistants from the Public Health Laboratory—Ivo de Carneri in Pemba. The local team had little or no previous experience with the application of qualitative research methods. Research assistants were trained in research ethics and qualitative data collection methods by the senior social scientist and served as the primary data collectors and logistic coordinators setting up focus groups and interviews within the communities.

### Recruitment of Participants

We used purposive sampling to recruit a homogeneous study sample of school-aged children, who might engage in risky behaviors. Such risk behaviors include swimming, fishing, bathing, washing clothes, or performing other domestic chores in ponds, lakes, streams, and rivers that are potentially contaminated with *S*. *haematobium* larvae. For this initial informative research study it was decided that the easiest way to reach school-aged children was through government supported public primary schools. Schools in the selected shehias were identified with the assistance of the staff from the Ministry of Health and from the Ministry of Education. Students in grades Standard 1 to Standard 7 were recruited through the headmaster of each school. Individual teachers and parents were also recruited through public primary schools and community leaders were recruited through local social networks.

### Data Collection

**Fig 1:**
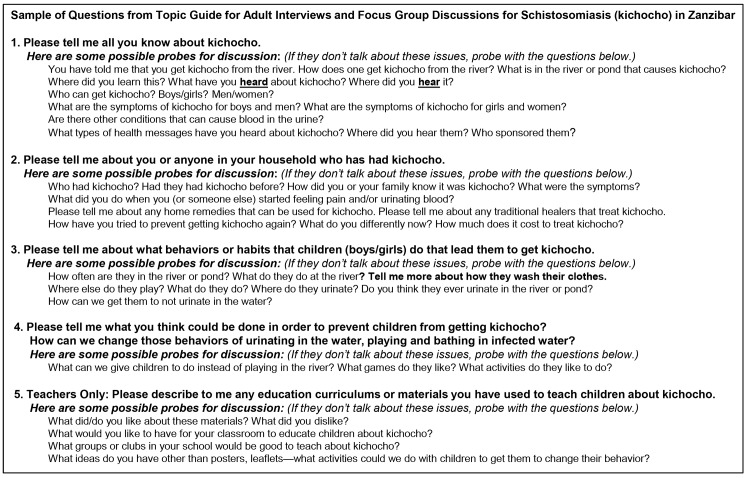
Sample of qualitative probes for adults participating in a qualitative study about schistosomiasis in Zanzibar.

The qualitative inquiry was conducted through 35 children’s group discussions (GD), 5 focus group discussions (FGD) with community leaders, and 41 in-depth interviews (ID) with teachers and parents. The children’s GD included 6–8 children of the same sex, facilitated by a Kiswahili speaking research assistant using a simple topic guide to lead the discussion (S1 Topic Guide). Children were provided paper and crayons and were first asked to draw anything they wanted to draw, followed by a discussion of their picture. Then they were asked to, *“Please draw me a picture about everything you know about kichocho (the local term for schistosomiasis)*.*”* These drawings encouraged more robust discussions [32]. Students drew pictures and then described their drawings about the disease, risk behaviors, and prevention ideas. A note taker managed an audio recorder and took written notes in support of the group facilitator. FGD with community leaders followed a similar format without the drawings. Individual IDIs were conducted with parents and teachers using the audio recorders without the note takers. Examples of qualitative open-ended questions used with the adults are shown in Fig 1. The study was conducted in five shehias on Unguja (Chaani, Dole, Kilombero, Mwera, and Uzini) and in two shehias on Pemba (Chambani and Kizimbani). The shehias were selected among the 15 behavioral study shehias on each island [19], based on their location on the island and previous knowledge about urogenital schistosomiasis in the area [29,31,33,34]. Data were collected until saturation was obtained [35,36].

All data collection tools including project overview, informed consents, and topic guides were translated from English into Kiswahili, pretested, and modified to adapt to local linguistic and cultural nuances by the research team [37]. Topic guides explored i) knowledge and perceptions of schistosomiasis transmission, ii) specific behaviors among children in Zanzibar that put them at risk for acquiring *S*. *haematobium* infections, iii) symptoms of schistosomiasis along with personal health-seeking behaviors and treatment strategies, along with iv) ideas to prevent schistosomiasis in children. Additionally, we explored other cultural factors, gender-roles, influential communication channels, and decision-making processes [37]. Additional probes allowed for deeper exploration of the topics that emerged, supporting additional areas of interest. Following group discussions and interviews, participants received a small thank you gift for their time and participation. Group discussions conducted in schools and FGD conducted in community settings were approximately 1.5 hours in length, IDIs took approximately 45–60 minutes. Data were collected using audio recorders (Olympus 70, Olympus Corporation, Tokyo, Japan). Additional field notes were handwritten during the interviews and reviewed during debriefing sessions to verify accuracy of the interview session [38]. We did not collect personal identifiers.

### Data Management and Analysis

Due to the paucity of behavioral information on urogenital schistosomiasis in Zanzibar we chose a qualitative approach to better understand cultural practices associated with activities of daily living linked with contact with local natural open freshwater bodies. In this study, we examined barriers to schistosomiasis prevention and control related to urination practices of children along with recommendations for improving such practices and reducing disease threats. We used a modified grounded theory approach with an emergent qualitative thematic analysis allowing the hypothesis to be generated from the data [39].

Narrative data were transcribed into English with review following translation to ensure accurate translation and local meanings. Transcripts were entered into Atlas-ti (ATLAS.ti Scientific Software Development GmbH, Berlin, Germany) as a Word document (Microsoft Corporation, Redmond, WA, USA) to facilitate text searching, data coding and analysis. Data analysis began with the first discussions and interviews allowing for emerging, unexpected, and/or inconsistent issues to be explored in subsequent discussions and interviews [36,39]. The coding structure evolved inductively with the codes from the narrative data of earlier interviews informing subsequent coding of the following interviews supplemented with field notes from the interviewer and note taker [32,38–40]. Due to time constraints and ongoing data collection tasks, the primary author, a social scientist experienced in qualitative research, was the primary data coder with verification of interpretive codes by the research assistants. Open, axial, and selective coding was used to analyze the GD, FGD, and IDI narratives [36,38–41]. A coding frame was developed through open coding, a word-by-word analysis used to identify, name, and categorize explanations and descriptions of the day-to-day reality of participants as related to schistosomiasis. Consensus on the coding frame was obtained through discussions with the local qualitative research assistants, who were from Zanzibar and conducted the original interviews. Axial coding, the process of relating codes to each other, via a combination of inductive and deductive thinking, was used for analysis of specific emergent themes, across themes, and for the relationships between themes [40,41]. Over the course of data collection, emergent themes became redundant, suggesting that all major themes had been identified and saturation reached [42]. An analysis matrix served as a framework for the resulting findings. Narrative excerpts from an analysis framework matrix are shown in Table 1.

**Table 1:** Narrative quotations from analysis matrix #2 to explore people’s perception of what causes schistosomiasis (kichocho).

Qualitative method	Quotations from individuals			
GD Older Boys; GD Young Boys	*We were told that kichocho is transmitted in the river*. *There are* *bacteria* *that cause kichocho*. *I think we have to educate our friends that kichocho is affecting the human body*. *K2;* **explore bacteria	*There are blood sucking worms*, *crabs and* *snails* *in the river that can all cause kichocho*. *M1*	*Kichocho is a disease that is transmitted if someone has gone to toilet and another person who doesn’t have kichocho steps there in bare feet*. *K3;* **common myth	*A person can get kichocho by* *eating chilies*. *Eating chilies causes kichocho*. *U2;* **many people have mentioned this
GD Older Girls; GD Young Girls	*Sometimes one may take water for brushing the teeth and when you* *gargle with the water* *there are organisms that will get into you*. *That’s how people get kichocho*. *U1*	*Kichocho is caused by the “bakari kichwa” (young frog)*. *There are also small fish that bite and cause kichocho*. *C2*	*Girls don’t get kichocho by going in the river*. *They are very few*. *The majority of girls get kichocho by* *eating chilies*. *M2;* **many people have mentioned this	*A* *snail bite**s you and you get sick with kichocho*. *K2*
Community Member FGD	*There are organisms in the river that may* *enter in the mouth* *from the way the children swim*. *They cause kichocho*. *U-FGD*	*Myself*, *I have never heard of it until I moved here*. *They told me it is transmitted by* *snails* *in the river*. *But I really know nothing*. *C-FGD*	*Kichocho is a disease that affects children and even adults when they go swimming in dirty water with small fish which cause kichocho*. *K-FGD*	*I don’t know if the exact cause of kichocho is the river or not*. *Maybe it is the* *water we drink*. *M-FGD*
Teacher IDIs	*You will get kichocho from organisms which live in* *snails*. *They penetrate through a person’s skin especially when you enter the water*. *D4*	*Most children get [intestinal] worms*. *This kichocho worm*. *It is different*. *I suffered kichocho when I was a boy as well*. *It is that way*. *C5*	*You know in some rivers and ponds there are* *snails* *that cause kichocho by producing small organisms that get into the human body causing the disease*. *P1*	*A person who baths in that river he will take on the kichocho disease*. *There is this* *bacterium* *in the river that causes kichocho*. *U3;* **explore bacteria
Parents IDIs	*Kichocho is a disease which is caused by dirtiness from the river*. *When people go to the river for their activities like washing clothes they make it dirty*. *C1*	*…the transmission can be between husband and wife*, *you know*. *K4*	*Kichocho can come from the soil or water or when the water is stagnant*. *These things they stick to the soles of your feet*. *M2*	*When you* *drink the water* *from the river you will swallow the tiny organisms and get kichocho*. *M1*

FGD: Focus group discussion; GD: group discussion; IDI: In-depth interview

The trustworthiness of our data was derived from standardization of methods and documentation for auditability, triangulation of the data, and verification of data findings with local staff members [43]. A standardized implementation document guided the training and implementation of the qualitative methodology with all procedures, topic guides, informed consents, timelines, interview schedules, data collection strategies, data management, and analysis strategies written out [38,43]. Triangulation of data was derived through the multiple data collection methods (GD, FGD, and IDI), multiple perspectives (younger and older girls and boys as well as adult women and men), and multiple venues (school-based, private home-based, and public venues). Findings were verified amongst local staff of the Ministry of Health in Unguja and the Public Health Laboratory Ivo-de-Carneri in Pemba by corroborating results with similar findings across other settings [44,45].

## Results

### Study Characteristics

As shown in Table 2, narrative data were collected from 27 children’s GD, 5 FGD with community leaders, and IDI with 21 teachers and 16 parents on Unguja Island. We also conducted 8 children’s GD and 4 IDI with teachers on Pemba Island to verify similarities among children on the two islands. Group discussions included boys and girls from both islands, who were 6–17 years old and attended grades of Standard 1–7. FGD included 16 male and 14 female community leaders, aged 23–72 years. They were teachers, farmers, leaders of women’s groups, religious leaders, school coaches, religious schoolteachers, petty traders, and small business owners, as well as housewives and traditional leaders. In-depths interviews were conducted with 2 male and 19 female teachers from Unguja and 2 female and 2 male teachers from Pemba. Teachers were 26 to 56 years of age. Eight fathers and eight mothers from Unguja were interviewed. Parents had a median age of 41years with a range of 24 to 72 years of age.

**Table 2:** Characteristics of groups involved in a qualitative study about urogenital schistosomiasis in Zanzibar.

	Children			Parents			Teachers			Community leaders		
	*Female*	*Male*	*Median age (range)*	*Female*	*Male*	*Median age (range)*	*Female*	*Male*	*Median age (range)*	*Female*	*Male*	*Median age (range)*
**Unguja**												
Group discussions (n = 27)	78	81	11 (6–17)				19	2	37 (26–56)			
Focus group discussions (n = 5)										14	16	46 (23–72)
In-depth interviews				8	8	40 (24–72)						
**Pemba**												
Group discussions (n = 8)	25	21	11 (8–15)									
In-depth interviews							2	2	35 (26–52)			

### Emergent Themes

#### Perception of Schistosomiasis transmission

Children and adults often identified “the river,” generic organisms, bacteria, small frogs, blood-sucking worms, crabs, dirt, other people’s urine, with few people reporting snails as part of the schistosomiasis transmission cycle. Young children often reported that leeches and small frogs caused kichocho. Older boys, many with previous experience of kichocho symptoms, reported receiving information about disease transmission from doctors, friends, or someone at school. An older male student said, *“We got this disease and when we went to hospital the doctor educated us about this so we can prevent it in ourselves*.*” (GD_K1)*. Despite this, most boys had a poor understanding about disease transmission. Children described several widespread myths about kichocho transmission. An older student said, *“Kichocho is a disease that is transmitted if someone has gone to toilet and another person who doesn’t have kichocho steps there bare feet*.*” (GD_K3)*. Another older girl also reported, *“Sometimes one may take water for brushing the teeth and when you gargle with the water there are organisms that will get into you… that’s how people get kichocho*.*” (GD_U1)*. Many teachers reported that they had limited knowledge of kichocho because there was only a small section in local science books. Teachers lacked a standardized, detailed syllabus to teach children. Only a couple of teachers had accurate knowledge about disease transmission, one reported, *“… you will get kichocho from organisms which live in snails*. *They penetrate through a person’s skin especially when you enter the water*.*” (Int_D4)*. Parents knew the least about the transmission and complications of disease. A couple of parents told us that kichocho could be transmitted man to woman during sexual intercourse. A parent said, *“…the transmission can be between husband and wife*, *you know*.*” (Int_K4)*. Overall, people perceived the infection as being insignificant, associated with worms that everyone has at one time or another. A male teacher told us, *“Most children get [intestinal] worms*. *This kichocho worm… I suffered kichocho when I was a boy as well*. *It is that way*.*” (Int*.*_C5)*

#### Risky practices of children

Kichocho was characterized by children and adults as a boy’s rather than a girl’s disease because boys spend more time in contaminated waters. Parents, teachers, and community leaders identified playing, bathing, fishing, and other water activities as risky behaviors of boys. A teacher reported, *“Small boys go there [to the river] with their parent…and others follow their friends in groups because they are told by their parent to go and bath at the river so they should not spoil water at home*. *Water is very little and difficult to get…*.*boys will bath for two hours or more in the river*.*” (Int_K3)*. A younger boy reported, *“When we go to the river it is fun*! *On Sunday there is no tap water so if you want to wash school uniforms you have to go there*. *When you go there you will pretend to put the clothes in the water but you are there to have fun in the water*.*” (GD_M2)*.

Most adults and some children associated the act of urinating in the river as a behavior that puts others at risk for disease. They described boys as urinating in the river more frequently than girls. Boys also told us that they urinated in the river, ponds, and fields. An older boy reported, *“Boys don’t like to urinate in the house*. *They urinate everywhere… even in the bushes*. *Men aren’t shy*. *Men will urinate anywhere*. *Especially the young ones*, *they will urinate in the water*, *whereas an elder hides when finding a place to urinate*.*” (GD_K2)*. Parents in every community acknowledged that boys urinate everywhere. A parent reported, *“When a boy is at home he uses the toilet*, *but if he is somewhere else he will urinate anywhere*. *I have been through all that*. *When I was a child I urinated in the river and everywhere*.*” (Int_D3)*.

Most adults reported that girls engage in fewer risky behaviors than boys. Teachers and parents told us that girls play in the river like boys until the age of 6 or 7 at which time they are separated from activities with boys, in line with their Muslim culture. Most risky activities were associated with domestic chores such as washing clothes and dishes as well as carrying water. An older girl said, *“…girls go swimming*, *wash clothes and dishes [at the river] and take water home for domestic chores*.*” (GD_C2)*. But another older girl told us that girls are not at risk for kichocho, *“They [girls] don’t void blood*. *They don’t get kichocho*.*” (GD_K9)*. Most adults, across all communities, described girls as using a toilet when urinating at home and urinating in the field when the need to urinate is urgent. A teacher told us, *“Girls urinate at their home and fields*, *very rarely in the rivers*.*” (Int_C2)*. Yet, some adults believed that girls do urinate in the river. A community leader told us that girls do urinate in the river, *“If girls are in the river they will urinate there*.*” (Int_M3)*. A young girl also told us, *“When we go to wash our clothes we urinate in the river*.*” (GD_U2)*. An older student also reported, *“Girls are different from boys*. *They may be a little shy*, *but they do urinate*, *but not like boys*. *Girls will hide in the water but boys will pour it openly*.*” (GD_M1)*.

#### Symptoms of kichocho

Most children and adults reported that kichocho symptoms were typically bloody urine with pain. An older boy reported, *“The urine will not come out and finally you will see blood coming out from the private part*. *You will get severe pain and at last there is a pus discharge and blood at the end when you finish*.*” (GD_K1)*. A parent with sons said, *“For kichocho there is severe pain at the kidney site*. *Sometimes urine may be leaking*, *itching*, *then bleeding*. *Signs are the same to boys and men but when the adult is sick he will go to the hospital himself*. *A boy will not say until the problem becomes chronic*.*” (Int_M4*).

Some adults and children reported uncertainty as to whether symptoms were different between adults and children and boys and girls. A teacher said, *“I am not sure if they [symptoms] are different between children and adults*. *I think they are all the same*. *I am not sure*. *I don’t know*.*” (Int_C4)*. A community member also reported, *“I think you feel pain at the urinary bladder*. *Also [there will be] blood in the urine because the disease is the same*. *The symptoms will differ according to the different nature of the male and female*. *Therefore*, *I don’t know which symptoms are for male and which symptoms are for female*.*” (FGD_D1)*. Adults and children failed to identify kichocho symptoms for girls and women. One parent reported, *“To them [girls] I think it would be very difficult to know [if she has kichocho]*. *For us*, *it is easy because we see it*. *However*, *for girls how would they know*? *Maybe when they wash their underwear and see blood they will know*.*” (Int_U3)*.

Community members we interviewed also provided a variety of other explanations for blood in the urine that could lead to delayed health-seeking behaviors. Many adults reported that gonorrhea, syphilis, and other sexually transmitted diseases, cancer, childbirth, and menstruation could cause blood in the urine. One man said, *“I think according to what we have heard*, *even a sexual transmitted disease like syphilis may cause blood in the urine*.*” (Int_Z1)*. In addition, parents, community members, and teachers reported that heavy exercise and exertion due to hard work could cause blood and pus in the urine. A teacher said, *“Sometimes strenuous exercises can cause blood in the urine*. *Don’t be surprised when a football player tells you*, *I have blood in the urine*. *I had this experience once*.*” (Int_MK1)*. A couple of parents and community members attributed blood in the urine to witchcraft: *“You can bleed if someone has done bad witch to you*.*” (Int_MW2)*. Several community members and parents and even a teacher attributed blood in the urine to eating local chilies.

#### Health-seeking behaviors and treatment

Many teachers, parents, and community members reported that while some people self-treat, most seek care at a hospital or clinic. Children were often reluctant to tell their parents they had kichocho. Costs of treatment varied, and even when free, people reported putting off seeking care because of transportation costs. Parents reported that they took children to local clinics only to find no available drugs for treatment. A referral to another facility, incurring more transportation costs, was a barrier to seeking treatment. A few parents, community members, and teachers described home remedies and traditional healers as first line care before going to seek care at a hospital for treating kichocho. Home remedies included preparing teas from the root of a plant or drinking copious amounts of water. A teacher reported, *“My relative used hospital treatment but there are some kind of roots which are used by people…*.*there are various roots for many diseases and kichocho can be treated by roots as well*. *There is one which is called “mkaanga uchawi” (frying a witch) or “mchafufu” some people call it a “tetracycline tree” because its fruits has two colors …red and black…..they boil the root and mix it with some herbs and drink the soup…it can be taken three times a day …it depends*.*” (Int_D4)*. A parent told us, *“I had painful urine and blood*. *I had signs like people were saying*. *I went to three traditional healers but it was useless…until I went to hospital and got better*.*” (Int_K2)*.

Mass drug administration in schools was often reported as the primary way children get treatment for kichocho: *“Children get drugs for free when health workers from Kichocho Program come to school*.*”* Parents described various experiences when seeking treatment for their children with kichocho through other avenues. A community leader told us, *“One has to go to hospital because sometimes local treatment does not help*. *You can be cured and get sick again so is better to be treated in hospital*.*” (FGD_D1)*. Another parent said, *“There was a time the drugs were free…now because of new technology one can go to private hospitals which treat Kichocho… you pay*.*” (Int_M6)*. There were many ideas about the costs of treating kichocho. Some adults and children reported that treatment was free. A parent said, *“We got treatment for free…we did not pay*. *It was good*.*” (Int_M7)*. But many others reported various treatment-associated costs. A teacher reported, *“The time I went [to the hospital] I got medicine freely*. *But transport cost from home to the center*: *going and returning*.*” (Int_Z2)*. A student reported, *“My friend was sick and we took him to the hospital where they gave him one tablet and he was asked to pay 400 shillings…one tablet*.*” (GD_K1)*. A teacher also told us, *“We buy [drugs for kichocho] in town at a private pharmacy*. *I remember a tablet cost about five hundred shillings*.*” (Int_C3)*.

Few adults knew where to obtain free treatment for kichocho. A teacher reported, *“I have heard that there is a special hospital for kichocho at Mianzini*. *I am not sure about the availability of drugs or if you have to buy or not*.*” (Int_D2)*. Parents also expressed concern over having to pay transport even if treatment was free: *“There are direct or indirect costs*. *An example of direct [cost] is buying tablets in private pharmacies or hospitals*. *An indirect [cost] is getting the tablets for free but paying bus fee to town*.*” (Int_Z1)*.

#### Children’s role models

Many adults identified parents, Madrassa (Koran school) teachers, and schoolteachers as role models for younger and older children. A parent said, *“Most of the time they [children] are with us*. *When you do something they follow*. *If it is good or bad…if you use abusive words they will do also*. *Girls follow the habit of their mothers and boys follow the habits of their fathers*.*” (Int_D4)*. Some parents reported that they were the most important role models for their children but that it was very challenging and they needed help to change their children’s behavior. *“We have been trying very hard without success*. *Maybe you can support us…we cannot make it happen*,*” said a parent*. *(Int_C2)*.

Many teachers reported that the parent is a critical role model for their child. Teachers also told us that support is needed to teach parents how to prevent their children from getting kichocho. A teacher told us, *“First it is the parents that are important*. *They need to get knowledge*. *A child should be educated about rivers at home so he can change behavior*. *We can also use the Sheha [community leader] committee members*. *They can fight against kichocho because they are respected by the community*.*” (Int_M8)*. Many other adults and also children acknowledged the role of a child’s peers in influencing their behaviors. An older boy from Kilombero shehia reported, *“The young ones [boys] they follow us*. *If they see us go to the river they will follow…even for fishing*. *So after a time he will get used to that and continue going to the river*. *If you say to him to stop going he will say*…*but you go*. *Why are you stopping me to go*?*” (GD_K3)*. A parent told us, *“A child will easily follow the behavior of another child*. *When he sees him doing something he will do the same*.*” (Int_M4)*. Additionally, it was acknowledged by most children that older boys are often the most influential role models, both positively and negatively, on the behaviors of younger boys.

#### Stopping urination in freshwater sources

Most adults and some children recognized the difficulty of extinguishing the behavior of urinating in the ponds and streams. It was seen as a private behavior and often associated with urgent need. A young student, when asked why he plays and sometimes urinates in the river when he knows not to reported, *“I am driven because of “ubilisi” (the devil)*.*” (GD_M2)*. Most adults interviewed suggested fear and punishment to prevent children from urinating in the river even though they themselves had been forbidden to go in the river and did so anyway when young. A parent told us, *“It is important to frighten them [children]*. *If you urinate in the river you will create and get diseases*. *If you urinate in the bush the devils will see you and you will be sick*. *We have to educate them to use toilet all the time even if they are outside*.*” (Int_M2)*. A community member reported, *“I think it’s simple to talk about but it’s difficult to change [behavior] because this boy in the bush*, *no one can see him [urinate]*.*” (FGD_D1)*. A teacher reported that a comprehensive educational effort was needed to change behavior, *“I think children need more health education*. *There should be street banners*, *more books on Kichocho in schools*, *and community mobilization like in malaria campaigns*.*” (Int_C2)*. Another teacher suggested going beyond lectures and encouraging more engaging activities for children, *“We can make small groups and teach them to prepare drama and role play to educate them about the effect of playing in the river*. *This would educate them not to urinate and bath in the river and ponds…..for children*, *the drama and role plays retain in their mind rather than words…..and this will help to change their behavior*.*” (Int_D3)*.

Children and adults across all groups interviewed suggested several structural interventions associated with changing the environment in order to facilitate behavior change in children. One teacher suggested, *“We should build toilets*. *The community should be educated about toilet building especially in public areas like schools*, *so children can learn toilet-using behavior*. *But we have to have toilets*.” *(Int_D4)*. A secondary student said, *“People can get together in a meeting and decide to dig a latrine and a well so we can get water which mixes with soap easily and use it to wash our clothes*. *They [children] won’t go to the river*.*” (GD_K2)*. Another secondary student suggested creating special policies, *“In our communities we do not use toilets*. *You should have a policy that everyone has to build a toilet in their home*. *It would help avoid using the water in the river*.*” (GD_C2)*.

#### Preventing kichocho in children

Community leaders, teachers, as well as students often described the need for community members to work together against kichocho. All groups of adults as well as students identified two major channels for reaching children not attending school. The first was reaching out and educating all parents in the community and the second was through Madrassa schools. One older student reported, *“This problem [kichocho] is affecting us so much that the Ministry of Health has to prepare a syllabus for us in schools*.*” (GD_C2)*. Another student suggested, *“I think we have to be friends with them [children] not to scold them*. *We should find the technique of making them to forget the river and to remind them to go to school and Madrassa*. *That would be better*.*” (GD_K3)*.

Teachers provided a variety of ideas for preventing kichocho. One teacher said, *“The first thing teachers need is training to provide knowledge to the children about everything about kichocho*.*” (Int_W2)*. Another teacher said, *“We need to create a syllabus concerning health and kichocho*.*” (Int_W1)*. Teachers also expressed a need for children to have alternative options for spending their spare time and playing. A teacher told us, *“We should organize interesting sports like football for students*. *Toilets and latrines should be built and children should be punished when they play in the river*.*” (Int_MK1)*. Parents also told us that children needed play options. One parent said, *“I think we should build playgrounds near the river so children will be attracted to play in the grounds instead of the river*.*” (Int_C4)*. A number of community leaders suggested improving water sources and increasing access to clean water for their community. A community leader told us, “*We can prevent kichocho by building as many wells as possible for clean water*.” *(FGD_C1)*.

## Discussion

Qualitative research, alone or in mixed methods, has been used to better understand the experiences of people affected or at risk for numerous neglected tropical diseases such chagas disease, filariasis, and schistosomiasis [46–48]. Results of our informative in-depth, qualitative investigation of schistosomiasis among school-aged children suggested that despite previous initiatives related to urogenital schistosomiasis control and prevention in Zanzibar [29,44,49], people’s knowledge about disease symptoms, transmission, and prevention were poor. Our findings identified several barriers to optimal disease prevention and control.

First, we observed that school-aged children regularly exposed themselves to contaminated natural, open freshwater bodies through recreational and domestic activities of daily living with little knowledge about routes of schistosomiasis transmission, which is in line with findings from previous studies in Zanzibar, Tanzania, Zimbabwe, and Western Kenya [50–52].

Second, *S*. *haematobium* infection was often viewed as an infection with an intestinal worm of little significance, not typically associated with severe health consequences, and little to no disease stigma. This is in contrast to reports from previous research in Nigeria, where individuals with schistosomiasis disease were stigmatized by others [53]. The Health Belief Model posits that perceived seriousness along with perceived susceptibility, perceived benefits, and perceived barriers are critical constructs used to explain and influence changes in health behaviors [54,55]. It also specifies that if individuals perceive a negative health outcome to be severe and perceive themselves to be susceptible to those negative outcomes, they are more likely to adopt positive protective behaviors [17,54,55]. Drawing upon the constructs of this behavioral theory supports shifting the context of schistosomiasis to that of a blood fluke, with serious health consequences such as bladder cancer and infertility, rather than the current perception of a less severe “worm.” Elaborating on the perceived seriousness of the infection, whether through medical information or increased awareness of the serious effects of the disease on a person’s life, is critical to address in a behavioral intervention [17,54,56]. There is evidence that theory-based, behavioral interventions can increase effectiveness among a variety of public health issues [57–60]. Synthesis of behavioral intervention research and non-regulatory interventions most often advocates the application of behavioral theory as an integral step in intervention design and evaluation [55,61].

Third, many people described abdominal pain, blood in the urine (hematuria), pain or burning during urination (dysuria), and commonly genital itching as symptoms of infection. However, as observed in studies conducted elsewhere in sub-Saharan Africa [62], these symptoms were also perceived as sexually transmitted infections that indeed may appear similar to symptoms of urogenital schistosomiasis. A person with a sexually transmitted infection may be reluctant to seek treatment due to shame and stigma [53,62]. Therefore, correcting the misperception that schistosomiasis is a sexually transmitted disease, while at the same time supporting the need to seek treatment for any and all similar symptoms, could be an important component of a schistosomiasis educational campaign to improve treatment seeking.

Fourth, first line treatment for a few people in Zanzibar, similar to mainland Tanzania [62], was often home remedies and occasional use of locally available herbalists followed by more conventional treatments when those earlier ones had failed. Lack of decentralized, locally available drugs and cost of transportation were also identified as barriers to seeking more conventional drug treatments. The decentralization of drug treatment to the local level as well as increasing knowledge about free drug treatment accessible through mass drug administration campaigns could improve treatment seeking among infected individuals. Further research into understanding any underlying barriers to treatment seeking behaviors should be explored [63].

Fifth, little available formal education about disease transmission contributed to myths and misperceptions about routes of transmissions, causes, and severity of disease, treatment, and ultimately prevention of disease. Schoolteachers and Koran school (Madrassa) teachers, viewed as influential people in children’s lives lacked formal scientific training, teaching materials, and other resources to be able to educate students about schistosomiasis. Teachers reported a need for a teacher’s training with a standardized, detailed syllabus to teach children about schistosomiasis during school sessions. Trainings could be set up similar to the Lushoto Enhanced Health Education Project that introduced interactive teaching methods into mainland Tanzanian study schools and demonstrated a feasible and effective intervention capable of changing schistosomiasis knowledge and health seeking behaviors among children [64]. The inclusion of religious teachers as change agents could maximize exposure of a schistosomiasis educational program to a broader community because they often engage children who may not attend government schools. Trained school and religious teachers could instill a perception of perceived seriousness of disease as well as perceived susceptibility of disease among children engaging in risky behaviors. Teachers could also identify and address the barriers to change and promote perceived benefits of reducing risky behavior to children.

Educating through schools could encourage students to act as change agents through peer education, role modeling, and shifting social norms of acceptable behavior [65,66]. Peer education, defined as “the teaching or sharing of health information, values and behaviors by members of similar age or status,” is widely used in the field of health promotion and education recently, such as the prevention of HIV/acquired immune deficiency syndrome (AIDS), smoking, and alcohol and drug use [67–71]. Peer education is focused on sharing information and experiences along with trust between the people in the similar context and learning from each other. Peer education, has been noted as a feasible method for transferring schistosomiasis knowledge from students to parents [65,66].

Sixth, most adults, and some children recognized the difficulty of extinguishing the behavior of urinating in the ponds and streams. It was seen as a private behavior and often associated with urgent need. Children and adults described educational, behavioral, and structural interventions to prevent kichocho in children. Community members often described the need for the community to work together to prevent kichocho in children suggesting the importance of a participatory approach to intervention development and implementation.

Previous research has noted that top down approaches to community interventions have been perceived by some community members as not in their best interest or being a poor fit for the socio-cultural context within the community [72–74]. The lack of attention to an individual’s social, cultural, religious, environmental, and physical context often results in a poor understanding of why an intervention is valuable and ultimately in an inadequate adoption of the desired positive behaviors and practices by community members [72–74]. This may explain why despite years of community administered preventive chemotherapy, the perception of schistosomiasis in Zanzibar was that of a commonplace, minor illness, rather than a serious threat to a child’s wellbeing. Administering preventive chemotherapy without addressing the local circumstances of community members with tailored communication and educational efforts can lead to not only misunderstandings but also to potentially poor treatment compliance [75,76]. Understanding community perceptions along with the social, religious, economic and environmental context of schistosomiasis risk and risk reduction behaviors among children can inform behavior change interventions that are relevant and provide meaning to the vulnerable populations in Zanzibar [74,77,78]. A recent evaluation of a comic-strip medical booklet *Juma na Kichocho* associated with a schistosomiasis health education campaign in 16 primary schools in Zanzibar reported disappointing findings [44,77]. The authors recognized that changing the behaviors of children could not be done by an isolated school curriculum but needed to consider their everyday realities of daily living [77]. The information garnered from our qualitative inquiry will allow for the ideas and problem solving solutions of community members to be incorporated into a behavioral intervention that is germane to others in their communities. Increasingly, there is a commitment to bringing a community perspective into research and implementation of interventions along with a growing body of evidence that public health and health-promotion interventions based on social and behavioral science theories are more effective than those without a theoretical foundation [55,79,80]. Drawing upon the constructs of perceived seriousness, perceived susceptibility, perceived benefits, and perceived barriers from the Health Belief Model complemented by a social ecological model that addresses multiple levels of the community could provide a functional framework for designing, implementing, and evaluating a health promotion program for the prevention and control of schistosomiasis tailored to the context of community members, particularly school-aged children [17,55,74,79,81].

There were several limitations to this inquiry. Given that we used a purposive, convenience sample, the findings may not be representative of all members of the communities in which the inquiry took place, and results are not generalizable. The triangulation of data suggests that there were similarities across behaviors of school-aged children attending the government primary schools on both islands where we conducted the student discussion groups. The behaviors we assessed appeared to be generally practiced among children across the shehias on Unguja and Pemba and the lessons learned could be used to tailor messages for future schistosomiasis control programs for primary school aged children. That being said, these findings are not generalizable to children attending private schools or not attending school at all. Further investigation is needed to explore the schistosomiasis knowledge, attitudes, perceptions, and practices of students in private schools and of students who do not attend school to assess if they are similar to those from children who participated in our student discussion groups. There may have been information bias during IDI, GD, and FGD as interview subjects may have provided answers that they believed the interviewers expected or wanted to hear. Additionally, bias may have been introduced due to only having a single coder, even though data interpretations and language translations were substantiated with local research assistants.

Conducting recreational and domestic activities of daily living in water contaminated with *S*. *haematobium* larvae compromises the health of school-aged children in Zanzibar. An important objective of this study was to facilitate improved design of an educational and control program. Urogenital schistosomiasis, characterized as a minor illness typically of boys, along with the lack of formal school-based and community-wide education about disease transmission, symptoms, and treatment can contribute to undiagnosed disease and a lack of treatment among both girls and boys. Understanding community perceptions of disease along with the barriers and facilitators to risk reduction behaviors among children can inform behavior change activities and health promotion programs augmented with chemotherapies for an integrated approach in support of the prevention, control, and elimination of urogenital schistosomiasis in Zanzibar and elsewhere.

## Supporting Information

S1 Topic Guide Zanzibar young students semi-structured topic guide for schistosomiasis (kichocho) drawing and discussion group. (PDF)
