# Supplementary material for: Community Knowledge, Perceptions, and Practices Associated with Urogenital Schistosomiasis among School-Aged Children in Zanzibar, United Republic of Tanzania
Source: PLoS Negl Trop Dis. 2016 Jul 11;10(7):e0004814. doi: 10.1371/journal.pntd.0004814 (PMC4939940; doi:10.1371/journal.pntd.0004814)
Supplement: S1 Topic Guide — (PDF) [file pntd.0004814.s001.pdf]

Zanzibar Young Students Semi-structured Topic Guide for Schistosomiasis (kichocho) Drawing and Discussion Group

Instructions

Younger students will be gathered together in small gender specific groups. Two team member will work with one group of younger children. Each team will have audio recorder, note paper for field notes, crayons and paper. There will also be a list for each table where the team member will write down initials, gender, ages, class. Please collect parent's informed consent prior to beginning. Introduce the audio recorder, turn it on and set it aside. Pause the recorder when the children are drawing.

Exercise 1-drawing and discussion

Please draw a picture of something you like to do. Something that you do that is fun! Please draw anything you would like to draw.
Remember this is to help the children become comfortable with drawing and describing the image. Team members should not act like parents. Please smile and encourage each child. Let them draw what they want to draw. Then ask 3 children to tell you the story about their drawings. Encourage them to talk. Note their story down in you field notes. Ask if anyone else would like to talk about their drawing. Encourage all children to talk about their drawing as practiced in our training session. Please collect the drawing but remember to tell the students they get to take these drawings home to their parents.

Exercise 2-drawing and discussion

You did such a nice job on the pictures you drew! We would love for you to draw another picture for us. Who wants to draw some more pictures? **How many of you have heard of kichocho? We would like you to draw a picture of everything you know about kichocho. You could show us how children get kichocho. Where you get kichocho. What causes kichocho. How you get kichocho. Please draw us anything you know about kichocho. And then we would like to hear all about your picture.** Remember to start the audio recorder and take any notes. When complete remember to probe using the questions below and any additional questions we used during the training.

Encourage students: *This is a lovely drawing. Please tell me about your kichocho drawing. Can you tell me more about your drawing?*
Here are some possible probes for discussion: *(If they don't talk about these issues, probe with the questions below.)*

> *Please tell me, what is kichocho? What do you think causes kichocho? Does anyone else have any other ideas about what causes kichocho?*
> *Please describe for me how you think children get kichocho? Please tell me about any other ways children get kichocho. Who gets kichocho?*
> *How do you know if you have kichocho? What happens if a child like you has kichocho? If someone is sick with kichocho what do they do?*
> *Where did you learn about kichocho? Who taught you about kichocho? Is there anything else you can you tell me about kichocho?*

At the end of the session please collect all of the kichocho drawings. Give out the drawing the children did earlier with the packet of biscuits and thank them for all of their great work! Collect all the equipment, drawings, informed consents, and files in their appropriate folders. Be sure to thank the teachers and headmaster. Nice job!
